# Transcriptome Sequencing and Positive Selected Genes Analysis of *Bombyx mandarina*

**DOI:** 10.1371/journal.pone.0122837

**Published:** 2015-03-25

**Authors:** Tingcai Cheng, Bohua Fu, Yuqian Wu, Renwen Long, Chun Liu, Qingyou Xia

**Affiliations:** State Key Laboratory of Silkworm Genome Biology, Southwest University, Chongqing, China; Institute of Plant Physiology and Ecology, CHINA

## Abstract

The wild silkworm *Bombyx mandarina* is widely believed to be an ancestor of the domesticated silkworm, *Bombyx mori*. Silkworms are often used as a model for studying the mechanism of species domestication. Here, we performed transcriptome sequencing of the wild silkworm using an Illumina HiSeq2000 platform. We produced 100,004,078 high-quality reads and assembled them into 50,773 contigs with an N50 length of 1764 bp and a mean length of 941.62 bp. A total of 33,759 unigenes were identified, with 12,805 annotated in the Nr database, 8273 in the Pfam database, and 9093 in the Swiss-Prot database. Expression profile analysis found significant differential expression of 1308 unigenes between the middle silk gland (MSG) and posterior silk gland (PSG). Three sericin genes (*sericin 1*, *sericin 2*, and *sericin 3*) were expressed specifically in the MSG and three fibroin genes (*fibroin-H*, *fibroin-L*, and *fibroin/P25*) were expressed specifically in the PSG. In addition, 32,297 Single-nucleotide polymorphisms (SNPs) and 361 insertion-deletions (INDELs) were detected. Comparison with the domesticated silkworm p50/Dazao identified 5,295 orthologous genes, among which 400 might have experienced or to be experiencing positive selection by Ka/Ks analysis. These data and analyses presented here provide insights into silkworm domestication and an invaluable resource for wild silkworm genomics research.

## Introduction

The wild silkworm *Bombyx mandarina* belongs to Lepidoptera *Bombycidae*. It is widely accepted as that *B*. *mandarina* is widely accepted as an ancestor of the domesticated silkworm, *Bombyx mori*. *B*. *mandarina* have been domesticated for at least 5000 years to increase and improve cocoon yield. Despite the short domestication history, many traits are different between *B*. *mandarina* and *B*. *mori* including body size and color in the larval stage, size and silk quality of cocoons, fight behavior and egg laying in the adult stage. Thus, *B*. *mandarina* and *B*. *mori* are good models for studying species domestication.

Recently, based on next-generation sequencing results, some studies have found that phenotypic changes are involved in genetic divergences. *Bombyx* resequencing identified about 16 million single-nucleotide polymorphisms (SNPs), 311,608 indels and 35,093 structural variations (SVs) in domesticated and wild silkworms. A total of 1041 regions, covering 2.9% of the genome and 354 protein-coding genes were detected with selection signals [1]. In addition, 347 SNPs were identified in the silkworm mitochondria genome. A cytochrome b gene shows strong positive selection in the domesticated group [2]. Copy number variation (CNV) is also an important domestication mechanism in silkworms. About 319 CNVs have been identified and about 49% are distributed on uncharacterized chromosomes. Approximately 61% of CNVs directly overlap with segmental duplications in silkworms. The genes in CNVs are mainly related to reproduction, immunity, detoxification and signal recognition [3]. For example, the copy number of carotenoid-binding protein (CBP) gene varies from 1 to 20 among *B*. *mori* strains. In contrast to *B*. *mori*, *B*. *mandarina* was found to possess a single copy of CBP lacking a retrotransposon insertion, regardless of habitat. The CBP gene is evolutionarily conserved in the lepidopteran lineage, showing that domestication can generate significant diversity in gene copy number and structure over a relatively short evolutionary time [4]. The domestication mechanism may involve regulatory elements. A putative expression enhancer located in the intron of the *Th* gene regulates transcription. The site might contribute to the body color transition from *B*. *mandarina* to *B*. *mori* [5]. In addition to genetic divergence, epigenetic divergence is also important in silkworm domestication. Comparative methylomics between domesticated and wild silkworms showed twice as many methylated cytosines in domesticated silkworms as in their wild counterparts, suggesting a trend toward increasing DNA methylation during domestication. Genes that are demethylated and have low expression in domesticated silkworms have experienced selective sweep, indicating a possible correlation with the enlargement of silk glands in domesticated silkworms [6].

Next-generation sequencing-based RNA-Seq analysis provides opportunities for *de novo* assembly of genome reference-free species [7]. This method provides information on gene expression, gene regulation and species evolution. Transcriptome analysis has been widely reported in some species such as *Formica exsecta* [8], *Delia antiqua* [9], and *Polistes canadensis* [10]. Recently, RNA-Seq has also been used as an efficient method to study adaptation to high-elevation environments or domestication. Based on *de novo* assembled transcripts and identification of orthologous genes, nonsynonymous site/synonymous site (Ka/Ks) analysis can provide insights into the process of adaptive evolution or domestication [11,12].

In the present study, we performed transcriptome sequencing for the middle silk gland (MSG) and the posterior silk gland (PSG) from *B*. *mandarina* using an Illumina HiSeq2000 sequencing platform. By *de novo* assembly, we generated a number of unigenes. By comparison with domesticated silkworm (p50/Dazao) genes and Ka/Ks analysis, we identified orthologous genes and genes that might have experienced or to be experiencing positive selection. Functional annotation of these genes provided information about the domestication mechanism in silkworms.

## Materials and Methods

### Sample collection and RNA extraction

Samples of wild silkworms were collected at the suburb (Lat/Lon: 29.60°N 106.28°W) of Chongqing city, China. No specific permissions were required for these locations. The field studies (Lat/Lon: 29.60°N 106.28°W) did not involve endangered or protected species. We dissected and collected two tissues: middle silk glands (MSGs) and posterior silk glands (PSGs) from a single fifth-instar larva. Tissues were immediately frozen and stored in liquid nitrogen. Total RNAs were extracted using TRIzol Reagent (Invitrogen) and treated with DNase. The quality and quantity of purified RNA were examined using Agilent Bioanalyzer 2100 (Agilent Technologies) and Qubit RNA Assay Kit (Invitrogen, http://products.invitrogen.com).

### Library construction for RNA-seq and sequencing

RNA libraries were constructed using Illumina TruSeq RNA preparation kits following the manufacturer’s instructions. Libraries were checked and quantified using Agilent Bioanalyzer 2100 (Agilent Technologies) and Qubit dsDNA BR Assay Kit (Invitrogen, http://products.invitrogen.com). Libraries were sequenced for 100 bp paired-end reads using the Illumina HiSeq2000 platform. Data analysis and base calling were performed by Illumina instrument software. Raw data presented in this publication have been deposited in the NCBI Short Read Archive (http://www.ncbi.nlm.nih.gov/sra/) and are accessible through SRA accession numbers SRX738967 and SRX738979.

### Sequence data analysis and *de novo* assembly

Adapter sequences were removed by Trimomatic 0.32 (http://www.usadellab.org/cms/?page=trimmomatic). Low-quality sequence reads were eliminated by scanning reads with a 4-base sliding window, cutting when the average quality per base dropped below 15[13]. Resulting sequence reads below 50 bp were removed. Before assembly, raw reads were assessed for quality using FASTQC (v0.10.1) software. We also removed ribosomal RNA reads by comparing with ribosomal RNA data collected using Bowtie2 [14]. *De novo* assembly of cleaned reads was carried out with Trinity software (http://trinityrnaseq.sf.net).

### Gene annotation

Open reading frames (ORFs) were predicted by the TransDecoder tool [15] using default parameters. Assembled unigenes were annotated using Trinotate annotation pipeline (http://trinotate.sourceforge.net/). Assembled unigenes were first used to BlastP alignment against the Swiss-prot database (downloaded 03/07/2014), using HMMER 3.1 [16] to identify protein domains by searching the Pfam_A database (downloaded 03/07/2014). Results were loaded using Gene Ontology (GO) terms classified with WEGO [17]. Assembled unigenes were also aligned to nonredundant (nr) proteins (http://www.ncbi.nlm.nih.gov/) and COG databases (http://www.ncbi.nlm.nih.gov/COG/). We used the online KEGG Automatic Annotation Server (KAAS http://www.genome.jp/kegg/kaas/) for KEGG pathway (http://www.genome.jp/kegg/) annotation with KO (KEGG Orthology) assignments. Pathway enrichment analysis of positive selective unigenes was performed with calculating methods as early reports [18]. Pathways with p-value ≤ 0.01 were defined as enriched.

### Analysis of gene expression

To analyze tissue-specific expression patterns, cleaned reads were mapped to assembled unigenes with Bowtie using run_RSEM_align_n_estimate.pl from Trinity software. RSEM 1.2.11 (http://deweylab.biostat.wisc.edu/rsem/) software was used to calculate expression levels as fragments per kilobase of exon model per million mapped reads (FPKM). Unigenes differentially expressed between MSG and PSG were detected with the edgeR bioconductor package [19]. If false discovery rate (FDR) was lower than 0.01, the p-value was lower than 0.05 and the highest FPKM of unigene was four times the lowest FPKM, a unigene was considered differentially expressed.

### Reads mapping and SNP identification

Cleaned reads were mapped to the assembled unigenes of the wild silkworm by Tophat2 (http://ccb.jhu.edu/software/tophat/index.shtml). SAMtools (tools for alignments in SAM format) software was used to detect putative SNPs [20]. Base quality was no less than 20 and coverage was no less than 5 for SNP detection.

### Analysis of positive selections

A reciprocal best hit method was used to identify putative orthologs between two species using BLASTP of BLAST-2.2.28+ [21]. We downloaded domesticated silkworm (p50/Dazao) CDS sequences from SilkDB V2.0 (http://silkworm.genomics.org.cn/). ORFs of assembled unigenes were compared to silkworm CDS sequences using BLASTP reciprocally to find orthologous pairs. Coding region of both sequences were aligned by Clustalw 2.1(http://www.clustal.org/clustal2/). A manual check was also conducted to correct potential errors. The ratio of the number of nonsynonymous substitutions per nonsynonymous site (Ka) to the number of synonymous substitutions per synonymous site (Ks) was used to test for positive selection. A Ka/Ks ratio greater than 1 was evidence of positive selection and a Ka/Ks ratio less than 1 indicated purifying selection [22]. KaKs_Calculator [23] software was used to estimate Ka, Ks and Ka/Ks ratio for each orthologous pair using the YN method [24]. Sequences with Ks >0.1 were excluded to avoid potential paralogs [25].

## Results

### RNA-Seq and *de novo* assembly

Libraries of MSG and PSG were sequenced using Illumina paired-end sequencing technology. In total, 120,381,200 raw reads were generated from wild silkworms (S1 Table). Removing adapters, low-quality sequences and ribosomal RNA yielded 100,004,078 clean reads with GC percentage 46.50% (S1 Table). Trinity software was used to assemble clean reads into 50,773 transcripts with N50 length 1764 bp and mean length 941.62 bp. Clustering generated 33,759 unigenes with N50 length 1437 bp and mean length 762.20 bp. Table 1 shows an overview of the assembled transcripts and unigenes.

**Table 1:** Summary of transcripts for *Bombyx mandarina*.

	Number (percentage)	
Length(bp)	Transcript (bp)	Unigene (bp)
**200–300**	14,829(29.21%)	12,428(36.81%)
**300–500**	11,338(22.33%)	8,488(25.14%)
**500–1000**	9,588(18.88%)	5,694(16.87%)
**1000–2000**	8,332(16.41%)	4,117(12.20%)
**2000+**	6,686(13.17%)	3,032(8.98%)
**Total length**	47,808,990	25,730,955
**Count**	50,773	33,759
**GC percentage**	39.91%	39.99%
**Median contig length**	478	374
**Average contig**	941.62	762.20
**N50 length**	1,764	1,437

### Sequence Annotation

For functional annotation, ORFs were predicted from assembled unigenes by the TransDecoder tool and annotated using Trinotate annotation pipeline with Pfam, UniProt/Swiss-Prot, and GO. We also annotated unigenes by aligning with Nr, KEGG, and COG databases for 12,805 unigenes significantly matched in Nr, 8273 matched in Pfam, and 9093 similar to proteins in the Swiss-Prot database (S2 Table). E-value distribution showed that 35.01% of Nr database annotated unigenes had an E-value equal to 0 (S1 Fig).

### Functional annotation

**Fig 1:**
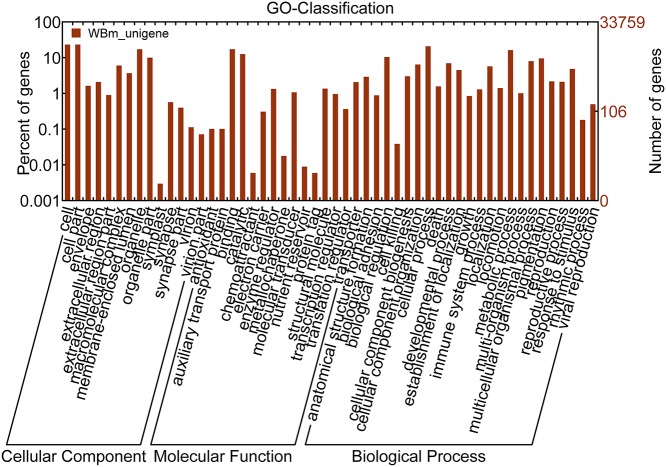
Gene ontology (GO) annotation.

GO categories are widely used to classify gene functions [26]. A total of 9571 unigenes had a GO annotation in three main groups (S2 Table, Fig 1). Within cellular component, 14 level-2 categories were identified. The top three groups were cell, cell part, and organelle. Within molecular functions, 15 level-2 categories were identified. The top three groups were binding, catalytic activity, and transporter activity. In biological process, 23 level-2 categories were identified. The top three groups were cellular process, metabolic process, and biological regulation.

**Fig 2:**
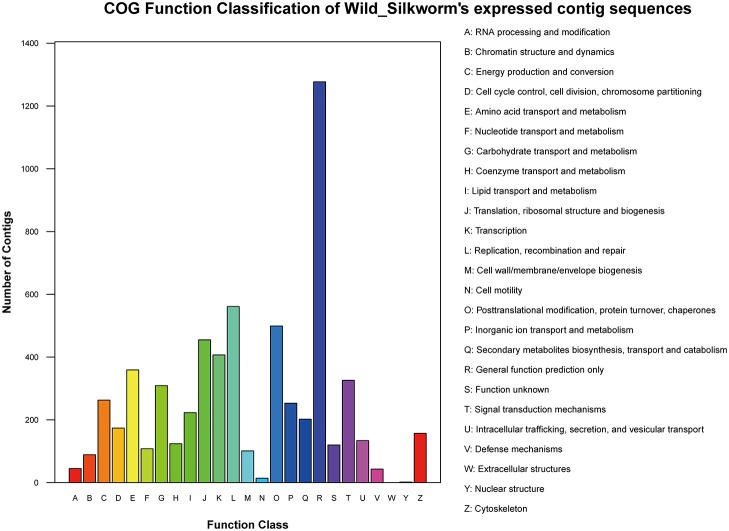
Clusters of orthologous group (COG) annotation.

A total of 6245 unigenes were classified into 25 functional categories (S2 Table, Fig 2). In addition to the largest group of general function prediction only (20.45%), the top four groups were replication, recombination, and repair (8.98%), posttranslational modification, protein turnover, chaperones (7.99%), translation, ribosomal structure and biogenesis (7.29%), transcription (6.52%), and amino acid transport and metabolism (5.75%).

A total of 5893 unigenes were assigned to 253 biological pathways in the KEGG database. Predicted pathways were divided into five biological classes (S2 Fig): organismal systems (29.66%), metabolism (24.90%), environmental information processing (17.53%), genetic information processing (14.77%), and cellular processes (13.14%).

### Gene expression analysis

A total of 1308 unigenes were differentially expressed (FDR ≤ 0.01, p-value ≤ 0.05, absolute value of logFC ≥ 2) between MSG and PSG in wild silkworms (S3 Fig) with 883 up-regulated expressed in MSG and 425 up-regulated in PSG (S3 Table). The silk gland is the only location that synthesizes and secretes silk proteins. The MSG synthesizes sericin and the PSG synthesizes fibroin proteins (Table 2). Three unigenes encoding sericin proteins were tissue-specific to the MSG. Four unigenes encoding fibrohexamerin-like proteins were tissue-specific with three unigenes showing significantly high expression. Tissue-specific unigenes were also involved in protein synthesis and secretion such as genes encoding silk gland factor 3, forkfead transcription factor G1, ABC transporters, and yellow proteins. In the PSG, three unigenes encoding fibroin proteins, fibroin/P25, fibroin-L chain and fibroin-H chain, were significantly specifically expressed. Two unigenes encoding glycine-tRNA ligase and alanine-tRNA ligase showed significantly high expression in the PSG. These gene products provide glycines and alanines for efficient synthesis of fibroin. Other specifically expressed unigenes encoded transcription regulation factors, heat shock proteins and sugar transporters.

**Table 2:** Summary of expression related to silk proteins between MSG and PSG.

WBm_unigene	length	FPKM_MSG	FPKM_PSG	logFC	PValue	FDR	Bm_CDS	Ka/Ks	Putative function
comp4655_c0_seq1	562	21.07	1580.95	6.035	1.29E-13	9.94E-12			Fibroin-H
comp11395_c0_seq1	1362	1298.87	137786.13	6.534	3.26E-15	3.24E-13	BGIBMGA009393	0.6473	fibroin-L
comp8545_c1_seq1	1353	182.40	31454.66	7.235	2.73E-17	3.76E-15	BGIBMGA001347	0.1938	fibroin/P25
comp14614_c0_seq5	2173	8339.87	6.45	-10.532	4.54E-27	4.99E-24			sericin 1
comp14333_c2_seq1	1846	7606.27	6.60	-10.366	1.51E-26	1.03E-23	BGIBMGA011901	1.2050	sericin 2
comp17080_c0_seq1	480	5175.15	6.27	-9.877	1.03E-24	4.19E-22	BGIBMGA012002	1.8625	sericin 3
comp13620_c0_seq1	3130	65.03	408.04	2.453	3.71E-04	4.75E-03	BGIBMGA006216		alanine-tRNA ligase
comp4656_c0_seq1	2624	121.56	986.25	2.825	5.35E-05	9.04E-04	BGIBMGA007637	0.0198	glycine-tRNA ligase
comp17114_c0_seq1	1334	530.01	0.43	-10.453	3.77E-25	1.74E-22	BGIBMGA009261		fibrohexamerin
comp5260_c0_seq1	1067	3.18	0.05	-5.883	1.63E-06	3.95E-05	BGIBMGA009261		fibrohexamerin
comp7638_c0_seq1	1509	1118.49	0.75	-10.727	1.15E-26	8.65E-24	BGIBMGA009261	1.6506	fibrohexamerin
comp8883_c0_seq1	954	24544.0	15.54	-10.819	6.09E-28	1.46E-24	BGIBMGA009261		fibrohexamerin

### Reads mapping and SNP calling

To evaluate heterozygosis in the wild silkworm genome, cleaned reads were mapped to assembled unigenes. A total of 32,297 SNPs and 361 INDELs were identified in assembled unigenes with a total length of 25,730,995 bp (Table 3) and with a density of 797 bp per SNP; 19,783 SNPs were located in ORFs, 4974 in 3'-UTRs, and 2024 in 5'-UTRs with a density per SNP of 586 bp for ORFs, 815 bp for 3'UTRs, and 811 bp for 5'-UTRs. Only 14 INDELs were identified in the ORFs, which was less than the 179 in 3'-UTRs and 54 INDELs in 5'-UTRs. Moreover, 16,074 (81.3%) SNPs in ORF induced synonymous amino acid substitutions (Table 3).

**Table 3:** Summary of SNPs and Indels for *Bombyx mandarina*.

	Length (bp)	SNP (num)	SNP_density (bp/num)	Indel (num)	Indel_density (bp/num)
**Unigene**	25,730,955	32,297	797	361	71,277
**ORF**	11,584,893	19,783/16,074/3,709 *	586	14	827,492
**Three_prime_UTR**	4,051,613	4,974	815	179	22,635
**Five_prime_UTR**	1,640,684	2,024	811	54	30,383

*Total number/ synonymous site number / nonsynonymous number

### Identification of putative orthologs and analysis of positive selection

A reciprocal best-hit method was used to identify putative orthologs between the two species [21]. A total of 5295 pairs of putative orthologs were identified by comparing the wild silkworm unigenes and the silkworm CDS sequences. The Ka/Ks ratio was calculated by KaKs_Calculator software showing 3855 pairs with Ka and Ks for calculating Ka/Ks and 1440 pairs with only Ka or Ks. The 3855 pairs had mean values of 0.0624 for Ka, 0.4845 for Ks and 0.6409 for Ka/Ks. Among 3855 pairs of orthologs, 1049 pairs had Ks > 1 and were considered potential paralogs. After removing potential paralogs, 2,806 pairs belonging to orthologs with mean values of 0.0109 for Ka, 0.0433 for Ks, and 0.7316 for Ka/Ks ratio (S4 Table).

**Fig 3:**
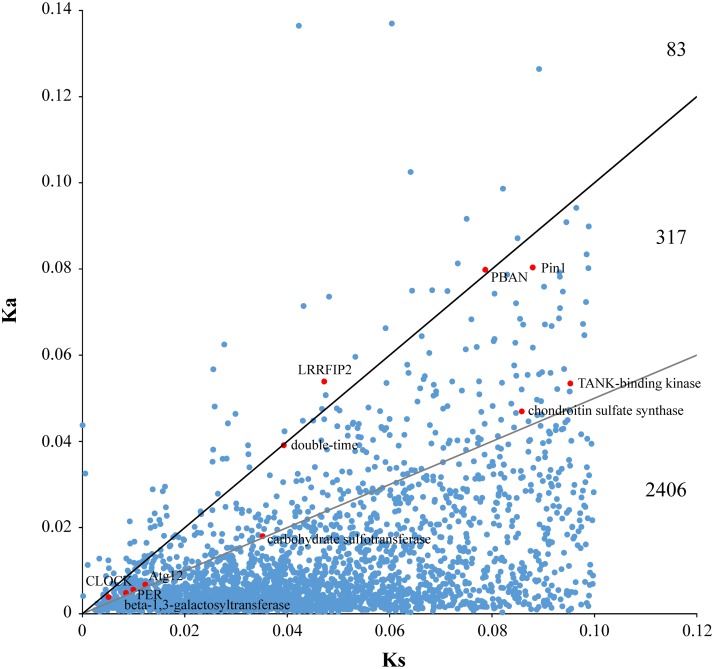
Distribution of Ka and Ks values. Above the black line, orthologous pairs with Ka/Ks ratio >1; between black and gray lines, pairs with Ka/Ks ratio 0.5–1.

A total of 83 (2.96%) pairs of orthologs had a Ka/Ks > 1 showing genes that might have experienced strong positive selection (Fig 3). A Ka/Ks ratio of 0.5 was considered as a useful cut-off to identify genes under positive selection [27], for 317 (11.30%) pairs of orthologs with a Ka/Ks ratio between 0.5 and 1 (Fig 3). Thus, 400 (Ka/Ks > 0.5) pairs were considered likely to have experienced or be experiencing positive selection. Comparing with 354 candidate domestic genes in genomic regions of selective signals (GROSS) from resequencing data [1], 13 genes were common between Ka/Ks and GROSS analysis (S5 Table). In addition, two unigenes (Ka/Ks = 0.68, 1.31) encoding cytochrome b-like proteins were considered to be under positive selection. The *cytochrome b* gene showed a strong signal of positive selection in the domesticated clade [2].

### Functional analysis for positive selection

**Fig 4:**
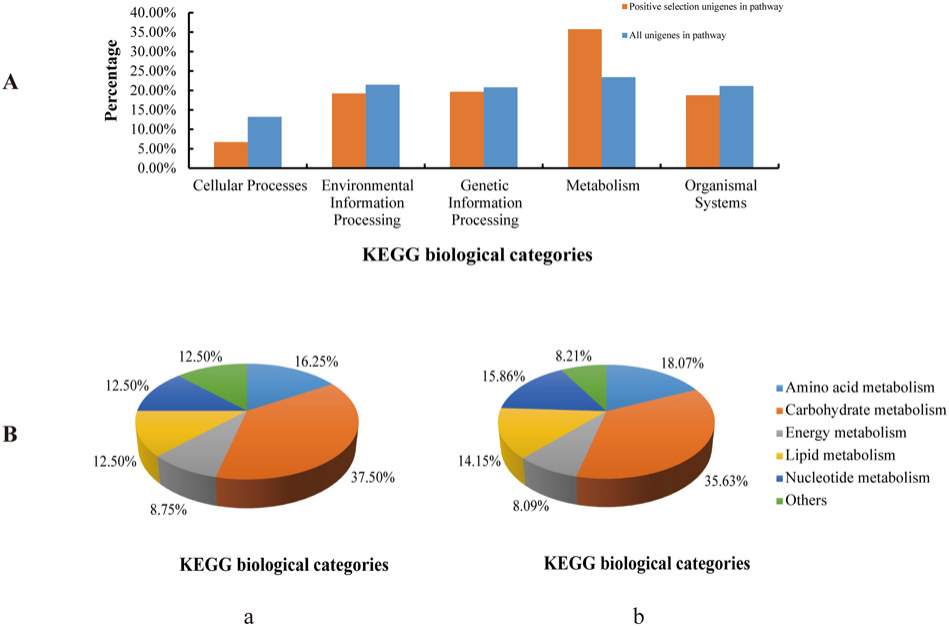
Distribution of KEGG biological categories of positively selected and all unigenes in pathways. (A) Distribution of five classes of positively selected and all unigenes in pathways; (B) Metabolism shows distribution of six classes. (a) Distribution of positively selected unigenes in pathways, (b) Distribution of all unigenes in pathways.

Among 400 unigenes with Ka/Ks >0.5 considered as positively selected, 168 were annotated with KO assignments (42%). KEGG pathway analysis was performed with KAAS for 126 KEGG pathways identified with unigenes (S6 Table). The unigenes were divided to five biological classes using pathway enrichment analysis. Fig 4A shows distribution of five classes between positive selection and all the unigenes in pathways. Only the class metabolism had a significantly higher percentage among positively selected genes than among all unigenes (35.71% vs. 23.43%). The metabolism class was approximately divided into six classes between positively selected and all unigenes in pathways: amino acid metabolism (16.25% vs. 18.07%), carbohydrate metabolism (37.50% vs. 35.63%), energy metabolism (8.75% vs. 8.09%), lipid metabolism (12.50% vs. 14.15%), nucleotide metabolism (12.50% vs. 15.86%), and others (12.50% vs. 8.21%) (Fig 4B). Pathway enrichment analysis identified three enriched pathways (p≤0.01): glycosaminoglycan biosynthesis-chondroitin sulfate/dermatan sulfate (ko00532), retinoic acid-inducible gene I (RIG-I)-like receptor signaling pathway (ko04622), and circadian rhythm-fly (ko04711) (Table 4).

**Table 4:** KEGG pathway enrichment analysis of positive selection.

KEGG pathway	Unigene	Bm_orthologs	Ka/Ks	Putative function
Glycosaminoglycan biosynthesis—chondroitin sulfate / dermatan sulfate (ko00532)	comp12639_c0_seq1	BGIBMGA007765	0.5734	beta-1,3-galactosyltransferase
	comp1835_c0_seq1	BGIBMGA003101	0.5113	carbohydrate sulfotransferase
	comp35605_c0_seq1	BGIBMGA012390	0.5474	chondroitin sulfate synthase
Circadian rhythm—fly (ko04711)	comp15415_c0_seq2	BGIBMGA007304	0.9945	double-time protein
	comp18252_c0_seq1	BGIBMGA000486	0.5633	period circadian protein (PER)
	comp6926_c0_seq1	BGIBMGA000498	0.7571	circadian locomoter output cycles kaput protein (COLCK)
RIG-I-like receptor signaling pathway (ko04622)	comp11847_c0_seq1	BGIBMGA003954	0.5546	autophagy-related protein Atg12
	comp12369_c0_seq1	BGIBMGA000198	0.5607	TANK-binding kinase
	comp6686_c1_seq1	BGIBMGA000679	0.9135	rotamase Pin1

## Discussion

In this study, we performed *de novo* transcriptome sequencing for *B*. *mandarina* using Illumina HiSeq2000 sequencing platform. We obtained more than 100 million clean reads and 33,759 assembled unigenes with N50 length 1437 bp and average length 762.20 bp. A total of 883 and 425 unigenes were up-regulated expressed in the MSG and 425 in PSG, respectively. We identified six unigenes encoding major silk components: three fibroin proteins (fibroin-H chain, fibroin-L chain, and fibroin/P25) and three sericin proteins (sericin 1, sericin 2, and sericin 3). These unigenes were specifically expressed in the MSG or PSG with similar expression profiles of their homologous genes in the domesticated silkworm [28]. *Fibroin-H* (FPKM = 1581; logFC = 6.04), *fibroin-L* (FPKM = 137,786; logFC = 6.53), and *fibroin/P25* (FPKM = 31,455; logFC = 7.23) were specifically expressed in the PSG, whereas *sericin 1* (FPKM = 8340; logFC = -10.53) *sericin 2* (FPKM = 7606; logFC = -10.37) and *sericin 3* (FPKM = 5175; logFC = -9.88) were specifically expressed in the MSG. An immunosorbent assay with specific antibody against each fibroin indicated that a molar ratio of fibroin-H chain, fibroin-L chain, and P25 of 6:6:1 [29]. However, the ratio in wild silkworms was 0.05:4.38:1. We propose that low expression of the *Fibroin-H* gene might be due to constraints of sequence technology for high GC-content and simple-repeat-sequence-containing genes. We also found unigenes involved in protein biosynthesis and secretion. Fibroin has high proportions of glycine (44.6%), alanine (29.4%) and serine (12.1%) [30]. For example, two unigenes encoding glycine-tRNA ligase (logFC = 2.82) and alanine-tRNA ligase (logFC = 2.45) were more highly expressed in the PSG than in the MSG, suggesting that the two genes participate in sericin and fibroin biosynthesis. Four unigenes encoding fibrohexamerin-like proteins were specifically expressed with FRPM values (24,544, 1118, 530 and 3.18) in the MSG. The silkworm genome has eight fibrohexamerin-like genes with *Bmfhxh4* expressed specifically in the MSG and involved in protein assembly and secretion [31].

The silk gland is one of the most important tissues that has undergone artificial selection for more and better quality cocoons. Many phenotypes have undergone changes between wild and domesticated silkworms such as silk gland size and silk output, and cocoon quality and color. By comparing with *B*. *mori* genes, we identified 5295 pairs of orthologous genes by Ka/Ks analysis, with, in among which 400 genes might have experienced or to be experiencing positive selection by Ka/Ks analysis. For instance, three genes encoding sericin 2, sericin 3, and fibrohexamerin-like experienced strong positive selection with Ka/Ks ratios of 1.21 for sericin 2, 1.86 for sericin 3 and 1.65 for the fibrohexamerin-like gene. *Fibroin-L* was found to be under positive selection (Ka/Ks = 0.65). In addition to these structural proteins, by KEGG analysis, several signaling pathways were involved in organ or tissue development and nutritional signals. A unigene encoding insulin (Ka/Ks = 1.12) has experienced strong positive selection. Only two SNPs were detected between wild and domesticated silkworms; both led to nonsynonymous substitutions (I^42^-V^42^ and S^110^-N^110^). Insulin is involved in many signaling pathways such as the insulin signaling pathway (ko04910), the mTOR signaling pathway (ko04150), and the FOXO signaling pathway (ko04068). In these signaling pathways, eight unigenes encoding an insulin growth factor 1 receptor (Ka/Ks = 0.79), a phosphorylase kinase (Ka/Ks = 0.62), a ras-related GTP binding protein (Ka/Ks = 0.53), a G-protein beta subunit (Ka/Ks = 0.86), a ubiquitin-like protein ATG12 (Ka/Ks = 0.55), a casein kinase 1(Ka/Ks = 0.99), a CREB-binding protein (Ka/Ks = 0.73), and a serine/threonine-protein phosphatase PP1 catalytic subunit (Ka/Ks = 0.58), have been experiencing positive selection. The insulin and mTOR signaling pathways are evolutionarily conserved in most eukaryotes and are crucial for systemic transduction of nutritional signals regulating cell growth and metabolism [32,33]. These genes and pathways have experienced positive selection, suggesting that they should be molecular targets of artificial selection in the domesticated silkworm.

Three pathways were enriched by KEGG pathway enrichment analysis, including glycosaminoglycan biosynthesis-chondroitin sulfate/dermatan sulfate (ko00532) (p = 0.006), RIG-I-like receptor signaling pathway (ko04622) (p = 0.007), and circadian rhythm-fly (ko04711) (p = 0.004). The chondroitin sulfate metabolic and RIG-I-like receptor signaling pathways are related to host immune response [34,35]. In the chondroitin sulfate metabolic pathway, three unigenes encoding a beta-1,3-galactosyltransferase (Ka/Ks = 0.57), a carbohydrate sulfotransferase (Ka/Ks = 0.51), and a chondroitin sulfate synthase (Ka/Ks = 0.55) have been experiencing positive selection. Chondroitin sulfate in the peritrophic membrane might protect the midgut epithelium from ingested pathogens [34]. In the RIG-I-like receptor signaling pathway, three unigenes encoded an autophagy-related protein Atg12-like protein (Ka/Ks = 0.55), a TANK-binding kinase (Ka/Ks = 0.56), and a rotamase Pin1 (Ka/Ks = 0.91) and are under positive selection. RIG-I-like receptors recognize RNA viruses and trigger a robust innate immune response against RNA virus infection [35]. The significant enriched two pathways might be a result of silkworm domestication against pathogens.

The domestication process also introduces diversity in behavioral phenotypes between wild and domesticated silkworms. For example, the domesticated silkworm moth has lost flight ability through artificial selection. A unigene (Ka/Ks = 1.14) encoding a leucine-rich repeat flightless-interacting protein 2 (LRRFIP2) has undergone strong positive selection. LRRFIP interacts with a leucine-rich repeat flightless protein that is a member of the gelsolin family and was discovered as a mutation accompanying disorganized flight muscle myofibrils in *Drosophila melanogaster* that leads to flightlessness [36,37]. Artificial selection targeting *LRRFIP* might be involved with loss of flight ability of the domesticated silkworm. The circadian rhythm pathway was reported to contribute to flight behavior in butterflies [38]. Three unigenes in circadian rhythm pathway that encode a double-time protein (Ka/Ks = 0.99), a period circadian protein (PER) (Ka/Ks = 0.56), and a circadian locomoter output cycles kaput protein (CLOCK) (Ka/Ks = 0.76) have been undergoing positive selection. Knocking-down the clock gene *period* leads to a small but detectable disruption in egg-hatching rhythm in domesticated silkworms, suggesting that the circadian rhythm pathway might influence egg-hatching behavior [39]. Domesticated silkworm eggs should be under artificial dark treatment so they hatch simultaneously in sericulture. Artificial selection pressure might lead to significant gene enrichment in the circadian rhythm pathway (p = 0.004). In addition, a unigene encoding a PBAN-type neuropeptide (Ka/Ks = 1.01) has also experienced strong positive selection. PBAN, a member of the pyrokinin family of neuropeptides, produces sex pheromone bombyxkol [40]. The positively selected gene might change the pattern of pheromones to affect sexual behavior in domesticated silkworms.

## Conclusions

In the present study, we performed *de novo* transcriptome sequencing analysis of *B*. *mandarina* tissues. More than 100 million reads were generated and assembled into 33,759 unigenes. A number of genes and pathways identified by Ka/Ks analysis may relate to artificial selection and domestication. A limitation of the current study is just the silkgland sample performed transcriptome sequencing. The transcriptome of other stages such as pupae and moths, or tissues such as midgut, fat body, and malpighian tubules might provide more information about immunity, metabolism and growing development during domestication. The data and analyses presented here provide insights into silkworm domestication and an invaluable resource for wild silkworm genomics research.

## Supporting Information

S1 Fig E-value distribution of *B*. *mandarina* transcriptome unigenes with annotation to Nr database. (PDF)

S2 Fig KEGG biological categories of *B*. *mandarina* transcriptome unigenes. (PDF)

S3 Fig MA plot and Volcano plot of differentially expressed unigenes (MSG vs. PSG). Red, differentially expressed unigenes: in MSG logFC < 0; in PSG logFC > 0.
(PDF)

S1 Table Summary of transcriptome sequencing for *Bombyx mandarina*. (XLSX)

S2 Table Annotation of unigenes. (XLSX)

S3 Table Summary of 1308 unigenes differently expressed between MSG and PSG. (XLSX)

S4 Table Ka and Ks of orthologous gene pairs between *B*. *mandarina* and *B*. *mori*. Ka: nonsynonymous substitution rate; Ks: synonymous substitution rate.
(XLSX)

S5 Table Summary of the positively selected genes identified between Ka/Ks analysis and GROSS. (XLSX)

S6 Table Summary of KEGG pathway enrichment analysis with positively selective unigenes (XLSX)
