# Supplementary figures and images for: Transcriptome Sequencing and Positive Selected Genes Analysis of Bombyx mandarina
Source: PLoS One. 2015 Mar 25;10(3):e0122837. doi: 10.1371/journal.pone.0122837 (PMC4373670; doi:10.1371/journal.pone.0122837)

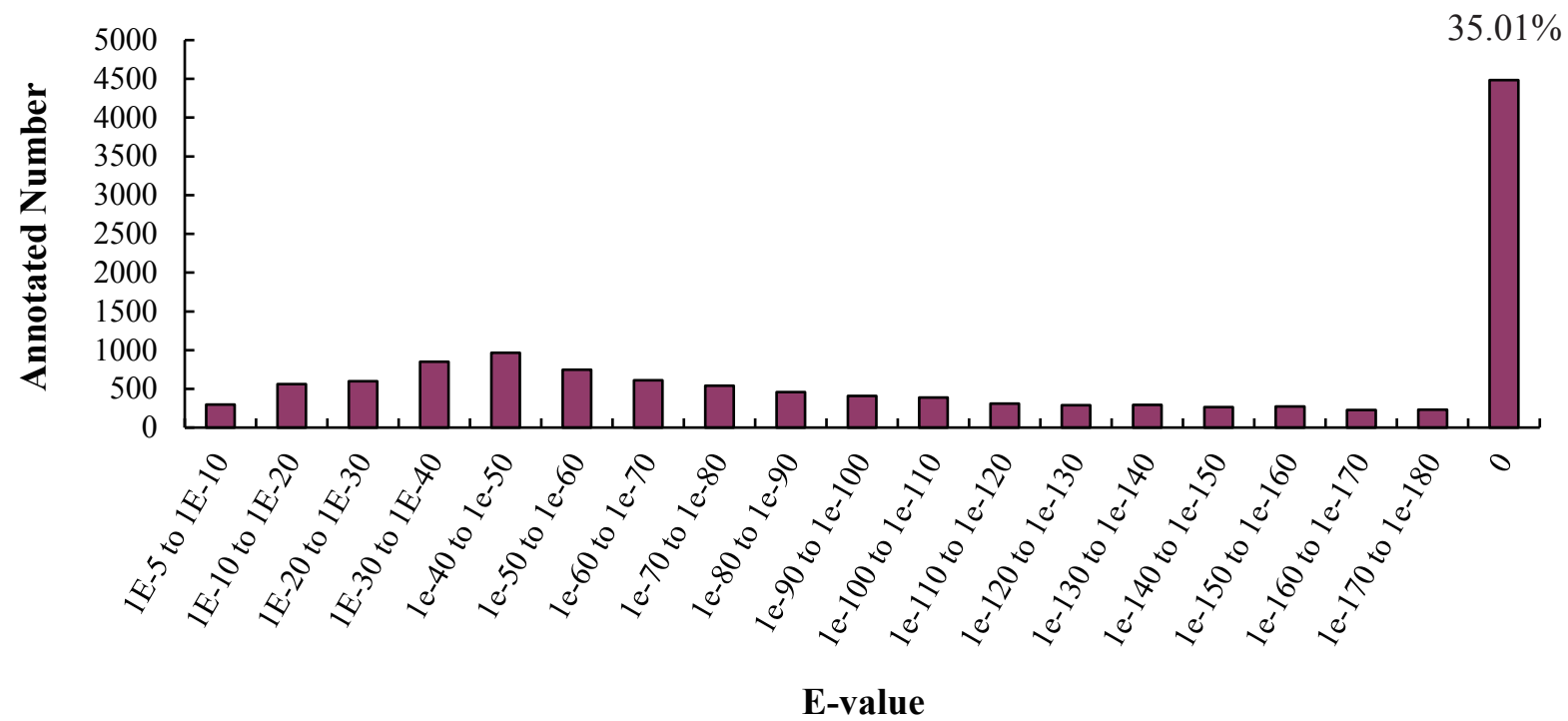

Supplement: S1 Fig — (PDF) [file pone.0122837.s001.pdf]

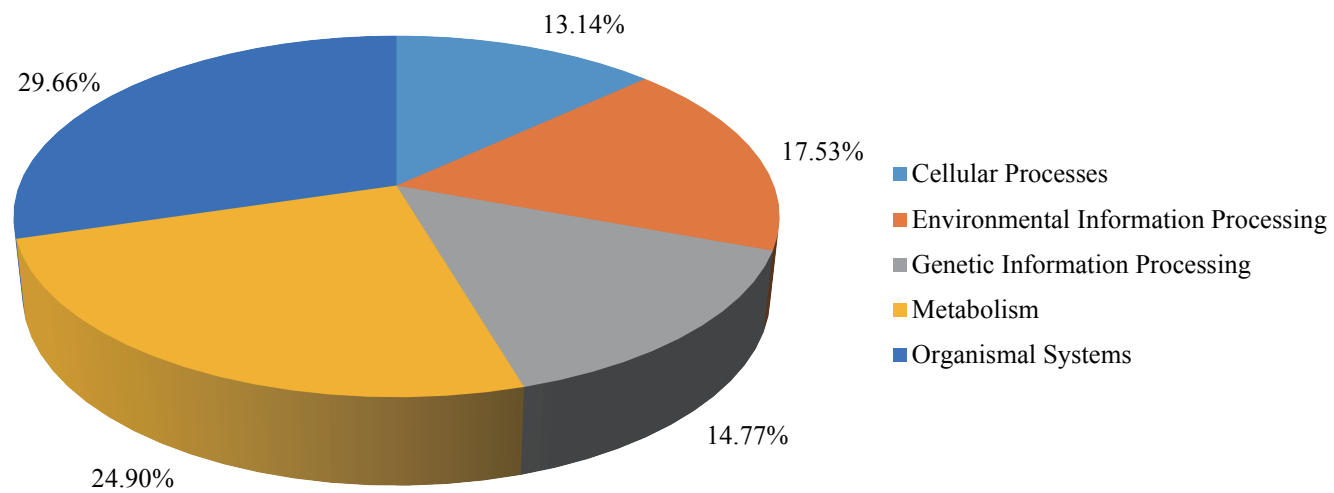

**KEGG biological categories**

Supplement: S2 Fig — (PDF) [file pone.0122837.s002.pdf]

**MA plot**

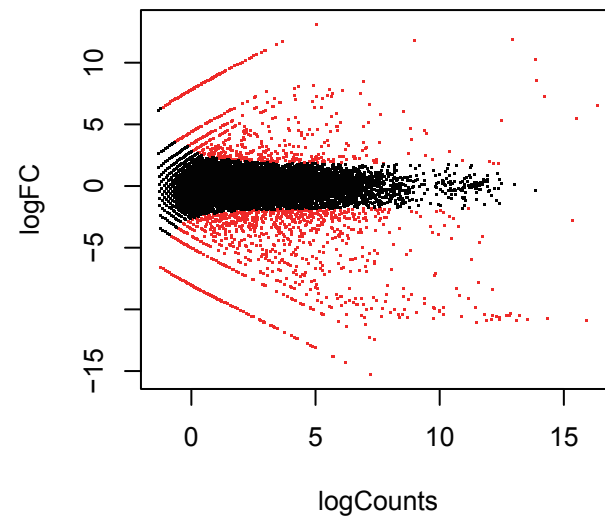

**Volcano plot**

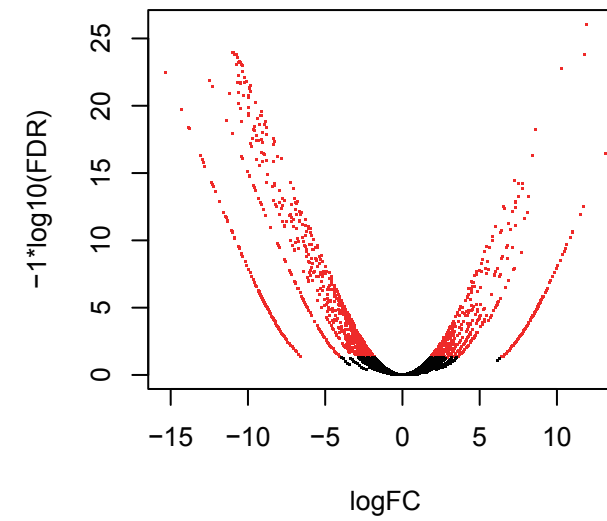

Supplement: S3 Fig — Red, differentially expressed unigenes: in MSG logFC < 0; in PSG logFC > 0. (PDF) [file pone.0122837.s003.pdf]
